# How the coronavirus pandemic affected the lives of people with ALS and their spouses in the UK from spouses’ perspectives: a qualitative study

**DOI:** 10.1080/21678421.2024.2346501

**Published:** 2024-05-08

**Authors:** Lyndsay Didcote, Ammar Al-Chalabi, Laura H. Goldstein

**Affiliations:** 1 Department of Psychology, Institute of Psychiatry, Psychology and Neuroscience, King’s College London, London, UK; 2 Department of Basic and Clinical Neuroscience, King’s College London, Maurice Wohl Clinical Neuroscience Institute, London, UK, and; 3 Department of Neurology, King’s College Hospital NHS Foundation Trust, London, UK

**Keywords:** Amyotrophic lateral sclerosis, caregivers, COVID-19, qualitative

## Abstract

**Objective:**

This study set out to investigate, using qualitative methodology, the experiences of spouses of people with Amyotrophic Lateral Sclerosis (ALS) during the coronavirus pandemic, with particular focus on spouse distress and cognitive and behavioral change in people with ALS (pwALS).

**Methods:**

Qualitative semi-structured interviews of nine spouses of pwALS living in England were conducted between 11/09/2020 and 20/04/2021, focusing on spouses’ perspectives of how their lives and the lives of pwALS were affected by the pandemic and related lockdowns. Interviews were subject to thematic analysis.

**Results:**

Four superordinate themes were identified from the spouses’ interviews: (i) pandemic behaviors, which encompassed accounts of cautious behavior, relaxation of cautious behavior, and other people’s attitudes to shielding the person with ALS; (ii) changes to daily life caused by the pandemic and progression of ALS; (iii) distress in spouses, which included anxiety, depression, and burden; and (iv) ALS-related behavioral impairment. Spouses also provided mixed accounts of telehealth care, pointing out its convenience but some felt that face-to-face appointments were preferable.

**Conclusions:**

While many reactions to the pandemic reported by spouses of pwALS may have been similar to those of the general population or other vulnerable groups, interviews indicated the potential for the pandemic to have made more apparent certain aspects of behavioral change in pwALS with which carers may require support. Clinicians need to acknowledge spouses’ concerns about the potential limitations of remote clinical consultations, enquire about cognitive and behavioral change, and consider how input should be best provided in such limiting circumstances.

## Introduction

The impact of pandemic (SARS-CoV-2) lockdowns on pwALS and their carers has attracted international research attention (1, 2).

The quality of life (QoL) of pwALS has been negatively affected by pandemic lockdowns (3, 4), potentially due to decreased social and environmental interactions (5). Pandemic-related isolation of pwALS is not always found to be problematic, however, because having ALS already leads to isolation (1).

In pwALS, feeling forgotten by clinicians or changed relationships with neurologists is associated with anxiety (6). Fear of being denied treatment in general has also been reported by pwALS (7). During the pandemic efforts were made by many clinics to continue to provide care remotely (e.g. via telephone or videoconferencing) to prevent disease complications, improve QoL for caregivers and limit pandemic-specific anxiety and depression (8).

However, healthcare for pwALS during the pandemic was considered sub-optimal by pwALS and healthcare professionals (9); this dissatisfaction reflected cancelations and delayed in-person appointments as well as replacement of in-person appointments with remote appointments. There were mixed views as to whether in-person appointments or a hybrid model of in-person and remote appointments was preferable (9).

Concern over the completeness of remote clinical assessments has been raised (4, 7) particularly over the provision of gastrostomy, respiratory function testing and noninvasive ventilation (7, 10). Nonetheless, pwALS report that telehealthcare reduces travel, costs, fatigue, and anxiety linked with COVID-19 infection (2).

Psychosocially, a study of pwALS and their caregivers found that caregivers report an increase in caregiving duties and related burden (1). A deterioration in the mental health of caregivers of pwALS and people with dementia (not including ALS-Frontotemporal Dementia; ALS-FTD) during the pandemic has been reported, with anxiety levels increasing (6, 11). Behavioral alteration in pwALS is one of the variables most strongly associated with caregiver burden (6).

National lockdowns (12) have resulted in ALS caregivers reporting feelings of isolation, loneliness, and helplessness, among other negative emotions; the greatest difficulty for caregivers of pwALS is the loss of support from healthcare professionals, increasing feelings of helplessness (12).

Between March 2020–March 2021, three national lockdowns were imposed in England by the government in response to the global COVID-19 pandemic (13). Given the impact of lockdowns on QoL, we set out to investigate, using qualitative methodology, (i) the experience of spouses of pwALS of lockdown in England in terms of methods of protecting themselves and how daily life changed; (ii) the impact of the coronavirus pandemic on spouses of pwALS in terms of spouse distress (anxiety, depression and burden); and (iii) the perceived impact of the coronavirus pandemic on pwALS in terms of progression of the disease, cognitive and behavioral changes and whether these made caregiving more difficult.

## Methods

### Participants

Spouses of pwALS who were aged at least 18 years old, and able to give informed consent were approached for this study. They had recently participated in other research by the authors estimating behavioral change in pwALS (and where the pwALS had themselves participated in a study on cognitive change in ALS). They were approached to minimize the likelihood of distress when contacting spouses of pwALS who were deceased. Participants were, therefore, known to the interviewer. Details about the interviewer (LD) are provided in Supplementary Information 1.

It was expected that 10–21 participants would be needed for this study before saturation of themes was reached, given that a purposive sampling method (Supplementary Information 2) was used and the scope of the measured phenomenon was small (14–16). Twenty-two spouses of pwALS were invited to participate (10 initially, with more being approached as others declined or were unavailable, until saturation of themes was reached); five people did not reply, and six declined the invitation. A total sample of 11 spouses of pwALS was recruited. However, due to technical issues only nine interviews could be transcribed and analyzed. Informal assessment during data collection indicated that saturation of themes had been reached.

### Data collection

**Figure 1.**
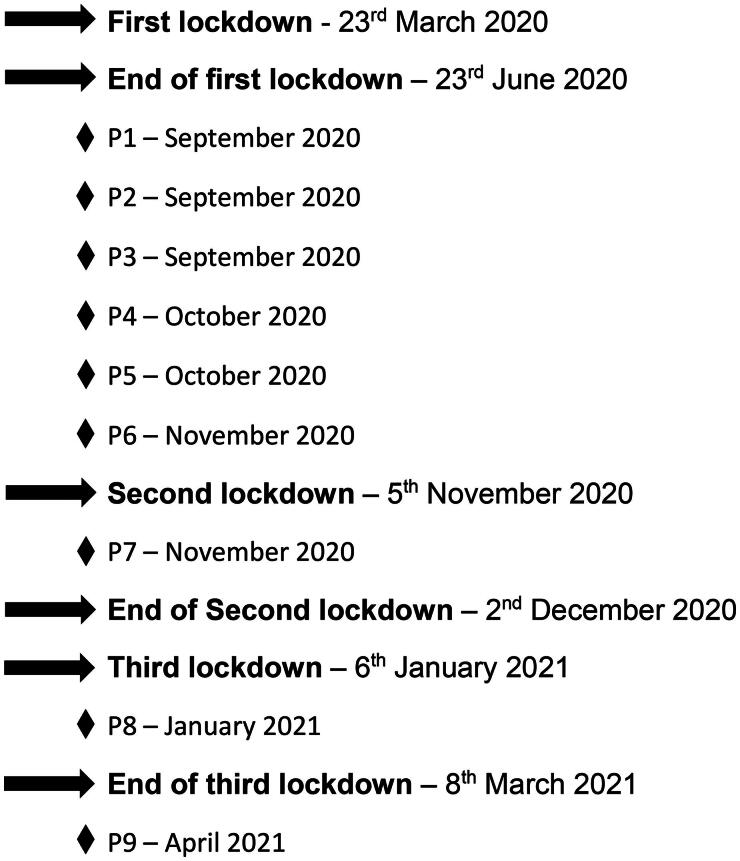
Timeline of interviews and coronavirus pandemic lockdowns in England.

Data collection occurred between 11/09/2020 and 20/04/2021 (Figure 1). Interviews were conducted online by LD during one session (50–80 min) using videoconferencing methods with participants who were at home. Semi-structured interviews were conducted using a topic guide. Only audio was recorded using screen recording software (AnyCap Screen Recorder V1.0.6.47 (17)). Further details about the conduct of the interviews are given in Supplementary Information 2.

### Topic guide and interview structure

The topic guide (Supplementary Information 3), developed by LD and LG, addressed the experience of lockdown from the perspective of spouses of pwALS and the impact of the coronavirus pandemic on pwALS and their spouses. If interviews occurred after the second and third lockdown in England, participants were asked to reflect on the first lockdown when answering questions.

Spouses were asked about the methods they and the person with ALS employed to avoid contracting COVID-19; how the pandemic affected the person with ALS in terms of daily routines, medical appointments and community support; and, to highlight potential unexpected benefits from lockdowns, what elements of daily life remained the same or improved over the lockdown period. Spouses were asked whether and how their caring duties, and levels of anxiety, depression and burden changed. Questions to spouses about how the pandemic affected the person with ALS were specifically related to physical symptom progression and cognitive and behavioral changes noticed because of the pandemic, and whether any identified cognitive or behavioral changes, if present, made dealing with the pandemic more difficult.

Questions were phrased to be open-ended, to avoiding leading questions. There was a focus on balance by asking for positive, as well as negative, effects of the pandemic. Probing techniques were used to cue participants to develop their answers; examples and anecdotes were encouraged. See Supplementary Information 2 for methods on guiding the interview.

### Data analysis

Thematic analysis was used to assess the interview data as it was the most suitable method for representing the views, opinions and experiences of spouses of pwALS. Supplementary Information 2 offers further justification for method choice and details of the analysis.

## Results

### Demographic information of participants

Participants were aged between 50–80. Six (66.7%) were female. All participants had known the person with ALS for over 10 years and had no other dependents. Seven participants (77.8%) did not report performing any caregiving duties for their spouse with ALS. Disease duration of the pwALS is shown in Table 1.

**Table 1.** Demographic information of participating spouses.

	Age group (years)	Sex	Reported caregiving duties?	Has professional caregiver	Hours caregiving by spouse per week	Relationship length (years)	Employment status	Disease duration of pwALS (years)
P1	60s	Female	Yes	No	2	11–20	Retired	2
P2	60s	Female	No	No	UH	31–40	Employed	1
P3	70s	Male	Yes	Yes	40	51–60	Retired	>6
P4	60s	Female	Yes	No	UH	31–40	Retired	5.5
P5	70s	Female	No	No	UH	51–60	Retired	1.25
P6	50s	Female	Yes	No	UH	31–40	Employed	3.33
P7	70s	Female	Yes	No	UH	41–50	Retired	5
P8	60s	Male	Yes	No	UH	31–40	Employed	3
P9	50s	Male	Yes	No	30	21–30	Employed	1.5

P = participant number. The length of relationships and ages are not given precisely to preserve anonymity. UH = under one hour per week.

For the spouse sample whose interviews were analyzed, the status of pwALS in terms of cognitive and behavioral impairment was varied (Table 2).

**Table 2.** Summary of cognitive and behavioral impairment in the associated pwALS according to various screening tools.

	Met cognitive impairment cutoff score				Met behavioral impairment cutoff score					
	CS 1	CS 2	CS 3	Any of the CSs	BS 1	BS 2	BS 3	BS 4	BS 5	Any of the BSs
pwALS 1							M		M	
pwALS 2					◊			◊		◊
pwALS 3						◊		◊		◊
pwALS 4		◊		◊						
pwALS 5										
pwALS 6		◊		◊			◊	◊	◊	◊
pwALS 7	◊	◊		◊				◊		◊
pwALS 8										
pwALS 9					◊	◊	◊	◊	◊	◊

pwALS = person with ALS. CS = cognitive screen. BS = behavioral screen. M = missing data. ◊ = pwALS met impairment cutoff score. The number assigned to each person with ALS is not related to the spouse participant number to preserve anonymity of participants. Cognitive and behavioral measures (the Edinburgh Cognitive and Behavioral ALS Screen (26), the ALS-Cognitive Behavioral Screen (27), the Mini-Addenbrooke’s Cognitive Examination (28), the Beaumont Behavioral Inventory (29), the ALS-Frontotemporal Dementia Questionnaire (30), and the Motor Neuron Disease Behavioral Instrument (31)) are not specifically named in the table to preserve anonymity of pwALS within the original dataset.

### Themes and subthemes

Four superordinate themes were identified: (i) pandemic behaviors; (ii) changes to daily life caused by the coronavirus pandemic and progression of ALS; (iii) spouse distress; (iv) ALS behavioral change.

Summaries of subthemes and codes within these subthemes that apply to pwALS specifically are presented in Tables 3–5, alongside example quotations from interviews. Quotations demonstrating other subthemes that might have been expected in the general population or other high-risk groups, might have been expected in caregivers of other groups, or might have been expected in pwALS had the pandemic not occurred, are presented in tables in Supplementary Information 4. Example quotations and results for the superordinate theme, pandemic behaviors, can also be found in Supplementary Information 4.

**Table 3.** Summary of identified subthemes and codes related to changes to daily life caused by the COVID-19 pandemic with illustrative quotations.

Subthemes	Codes	Example quotes
Changes due to COVID-19 pandemic	Changes to medical appointments	3.1.1.1 “The speech therapists does it on the phone.” P1
		3.1.1.2 “I mean he’s probably quite happy not maybe having to go into the hospital in a way.” P2
		3.1.1.3 “I don’t understand now why they don’t want to see her, why they want to keep doing this sort of uh sort of um Teams meetings type… it’s like they… it’s easier for them and they can get more done, I guess they’ve got a backlog but from a patient point of view, I don’t think it helps PATIENT… mentally” P9
Changes caused by an interaction between ALS and the pandemic	Losing the opportunity for experiences	3.2.1.1 “So PATIENT is missing out on golf… which is very frustrating for him because as his arm is getting weaker… it is hard for him to know that the longer it goes on for, the less likely it is for him to be able to play.” P7
		3.2.1.2 “clearly frustrating at a time when she is mobile, she’s not able to go and do the things and go on holidays and stuff that we want to.” P8
		3.2.1.3 “I feel a little bit sort of robbed… when you feel that you- your time might not be that long, it’s it’s unfortunate that you know, we could have had nice holidays and nice weekends and things.” P6
	ALS is a lockdown	3.2.2.1 “Because of PATIENT’S problems, it wasn’t as though we were leading a full life and then suddenly the pandemic and we had to alter our way of life… we just carried on as we were.” P3
		3.2.2.2 “I sort of think about being involved with the disease a lockdown itself… Now I am in a permanent lockdown because of the disease, not just the pandemic.” P1

Ellipses indicate where words have been omitted from the quotation for the purpose of representing the meaning of the quote within a limited space. P = participant number. ALS = Amyotrophic Lateral Sclerosis.

**Table 4.** Summary of identified subthemes and codes related to distress in spouses as a result of the coronavirus pandemic with illustrative quotations.

Subthemes	Codes	Example quotes
Anxiety	About the pwALS contracting COVID-19	4.1.1.1 “It was anxious I would say, anxious… Um well my concerns are that if he gets the virus, it will affect his breathing… it would be difficult for him to cope with.” P5
		4.1.1.2 “I probably feel more concerned than he does because I just don’t want him to get it and don’t want you know for me to get it either” P2
		4.1.1.3 “sometimes, as I've touched on before, I think I I'm more anxious about bringing something back to PATIENT.” P4
Depression	Loss of interest in doing things	4.2.1.1 “some days I have felt sitting around, even just reading a book. I have felt I need to do something to lift me – not lift me but do something to about it.” P5
		4.2.1.2 “some days, I feel less like getting up in the mornings than others.” P4
		4.2.1.3 “the garden… had just got to peek… perfection… so now I've got to do this one and… I'm less physically able to but uh I, I, yes, I think I'll, I'll get my interest back.” P4
	Sleep disturbance	4.2.2.1 “I think sometimes I I find it difficult to get off to sleep.” P6
		4.2.2.2 “I feel, when I go to bed at night I feel all calm and relaxed. And my head hits the pillow and my mind switches on. And I can still be awake still at 2am.” P1
		4.2.2.3 “I wake up more often during the night and think about things that probably won’t ever happen. So yes my sleeping pattern has been more erratic.” P5
	Negative thoughts	4.2.3.1 “I think it’s given me more time to think about our situation… that’s been the hard the hard bit… it’s just having more time to let things invade your thoughts.” P6
	No increase in depression	4.2.4.1 “I don’t think it has really made me feel more depressed.” P9
		4.2.4.2 “No, not at all. I’ve never suffered from any mental problems.” P3
Burden	Increase in burden	4.3.1.1 “there are some days where I would like someone to look after me” P5
		4.3.1.2 “from my knowledge or lack of it, yes I feel burden has changed because I don’t feel I am getting any, the information I need for now or certainly for the fast progression that this disease is likely to have.” P1
		4.3.1.3 “may be better because I won’t have to think about getting her to work and stuff… having someone else look after her and which allows me to leave.” P9
	No burden	4.3.2.1 “Um well, I don’t I don’t feel um that I'm a carer really yet. Um, so I don’t feel there’s a burden there.” P6
		4.3.2.2 “No. Not at all, just carried on the same.” P3
		4.3.2.3 “Um I don’t know if the pandemic’s changed that really” P4

Ellipses indicate where words have been omitted from the quotation for the purpose of representing the meaning of the quote within a limited space. P = participant number. pwALS = people with Amyotrophic Lateral Sclerosis.

**Table 5.** Summary of identified subthemes and codes related to ALS behavioral change with illustrative quotations.

Subthemes	Codes	Example quotes
Behavioral change noticed by spouse	Emotional lability	5.1.1.1 “someone doing well in a quiz program that he always watches. If they did well, I noticed he would start welling up and having to control himself.” P1
	Apathy	5.1.2.1 “He is not wanting to get involved in new things. You know, activities I have suggested… He does a lot of television watching, which is something that has definitely changed.” P1
		5.1.2.2 “like ‘I don’t see the point of going for a walk if um it’s not for a reason, to walk a dog or something’ so I thought, well OK I'll just go on my own then.” P4
	Denial or lack of insight	5.1.3.1 “PATIENT got carried away with moving as if nothing was wrong with her… She sometimes doesn’t… she forgets she has to do things differently.” P9
	More detail oriented	5.1.4.1 “And making spreadsheets of the electricity and gas meter. He has always been slightly interested in spreadsheets but in a much bigger form.” P1
Behavioral change that directly affects spouse	Abrupt communication	5.2.1.1 “When he does give advice, it sounds like quite abrupt almost criticism. I know it isn’t meant to be but it’s just his ability to communicate now does sound abrupt.” P1
	Egocentric behavior	5.2.2.1 “she is very focused on what she is doing to the extent that sometimes I’m not sure she is thinking about what anyone else what I am doing. It’s even like I am invisible sometimes.” P9

Ellipses indicate where words have been omitted from the quotation for the purpose of representing the meaning of the quote within a limited space. P = participant number.

### Changes due to ALS or due to the pandemic

Three subthemes were identified: changes due to progression of ALS; changes due to the coronavirus pandemic; and changes caused by an interaction between ALS and the pandemic. Example quotations for changes due simply to the progression of ALS are presented in Supplementary Information 4.

*Changes due to the coronavirus pandemic*. This theme was formed of three codes: change in caregiving duties (Supplementary Information 4); changes to social interaction (Supplementary Information 4); and changes to medical appointments (Table 3). Most spouses had observed digital appointments with clinicians for the person with ALS and some experienced delays and cancelations of appointments with neurologists. The benefits of remote appointments included not visiting the hospital, which saved time and money; less formality; and the person with ALS did not need to use public transport which was helpful in preventing COVID-19. Four participants felt there was no difference between in-person and digital appointments and this gave them more options. However, five participants doubted the ability of clinicians to make accurate assessments of pwALS via videoconferencing, particularly assessments that traditionally involve clinicians touching their patient.

Of note, one spouse expressed concern that in-person appointments were canceled at short notice and poor aftercare was provided following a remote ALS diagnosis. They felt that the subsequent digital appointments were more organized but left little time for reassurance from clinicians and this affected the mental health of the person with ALS (quote 3.1.1.3, Table 3).

*Changes caused by an interaction between ALS and the pandemic.* Participants often spoke about the challenges of separating the pandemic from the progression of ALS when trying to describe what had caused their lives to change. Disease progression and pandemic restrictions seemed to interact. Pandemic-related restrictions prevented pwALS from undertaking their hobbies or traveling. Over the pandemic period, their physical condition had deteriorated to the extent that, when pandemic restrictions were lifted, they would no longer be physically able to pursue these. Five participants felt that the period of time over which the pandemic had occurred had been the last chance for the person with ALS to have emotionally valuable experiences and this time had now been lost (quote 3.2.1.3, Table 3).

Two participants struggled to think how their lives had changed during the pandemic because of the restricted way in which they lived to accommodate symptoms of ALS. One participant described ALS itself as a lockdown (quote 3.2.2.2, Table 3). No spouses perceived an increase in the progression of ALS symptoms due to the pandemic. However, one participant spoke about the pandemic preventing the person with ALS, who was dysarthric, from being able to talk frequently with others which damaged their confidence and led them to avoid social interactions.

### Distress in spouses

*Anxiety.* Anxiety was experienced by most participants and was predominantly about the person with ALS contracting COVID-19 (Table 4), the spouse contracting COVID-19, or being in crowds. Some reported no anxiety (Supplementary Information 4).

*Depression.* Spouses primarily reported a loss of interest in doing things and sleep disturbance (Table 4). Participants also commented on changes in how they felt about themselves, feeling emotional or sad, experiencing negative thoughts (quote 4.2.3.1, Table 4), and having a limited capacity to worry about the person with ALS. Four spouses did not report any change in terms of frank depression, though most reported mild changes.

*Burden.* Only three participants reported increased feelings of burden (see Table 4 for examples). One participant who expressed an increase in burden described it as resulting from a lack of professional support during the pandemic (quote 4.3.1.2, Table 4). Others felt that the pandemic had not caused them to experience more burden or reported that they were not yet a caregiver and so felt no associated burden.

### ALS behavioral or cognitive change

According to the spouse participants, none of the pwALS had received a clinical diagnosis of behavioral impairment, cognitive impairment, or ALS-FTD.

*Behavioral change noticed by spouses.* Three participants (P1, P4, P9; Table 5) noticed behavioral change in the person with ALS that had occurred since the beginning of the pandemic. The most common change was apathy; emotional lability and becoming more detail oriented, denial or lack of insight were also reported. Apathy was perhaps noticed by spouses during the pandemic because usual activities for the person with ALS were disrupted and they had no interest in finding something else to do with their time, particularly the activities suggested by the spouse (quote 5.1.2.1, Table 5).

*Behavioral change that directly affects the spouse.* Two spouses reported behavioral change in pwALS that impacted their interactions with them (P1, P9; Table 5). One participant reported an increase in abrupt communication, although they questioned whether this was related to dysarthria rather than reflecting behavioral change. The other reported an increase in egocentric behavior which included ignoring the spouse to the extent that they felt “invisible”, and they described this behavior as “bizarre” (quote 5.2.2.1, Table 5). If participants had noticed changes in the behavior of the person with ALS since the beginning of the pandemic, they did not believe that the pandemic had influenced these changes. None of these participants reported that behavioral changes in the person with ALS made dealing with the lockdown more difficult. None of the spouses reported any cognitive changes in the person with ALS.

## Discussion

We identified four superordinate themes from the accounts given by spouses of pwALS: (i) pandemic behaviors; (ii) changes to daily life caused by the coronavirus pandemic and progression of ALS; (iii) spouse distress; (iv) ALS behavioral change. These superordinate themes are consistent with themes identified from surveys of pwALS concerning the pandemic (4). Aspects of their reactions may resemble those seen in the general population or other vulnerable groups (Supplementary Information 4).

What is particularly noteworthy is how the restrictions in place to prevent the spread of COVID-19 were reported to have interacted with disease progression over the pandemic period to result in a sense of lost time; pandemic restrictions prevented life-enriching activities and mobility declined at such a rate that when pandemic restrictions were lifted, it would have been impossible for pwALS to physically participate in those life-enriching activities. ALS participants in another qualitative study (1) hinted at this when they reported not being able to visit and socialize with loved ones during the pandemic, feeling that they had limited time to do this before deterioration prevented them from so doing.

The other particularly important finding is that some spouses felt their lives had not changed at all, even in full lockdown conditions, because their lives were already so restricted by the disease. Previous qualitative studies have similarly reported that isolation due to the pandemic was not problematic because pwALS and their caregivers were already isolated due to ALS symptoms (1, 18).

Views about digital medical appointments were mostly positive, with comments focusing on the reduction in costs, stress and time associated with traveling to appointments, supporting other findings (1, 2, 11, 19). Concerns were primarily over the ability of clinicians to make accurate remote assessments of physical symptoms.

Anxiety was frequently reported by spouses in this study, as found elsewhere in caregivers of pwALS (6). Feelings of anxiety reported by the current spouses were often connected to the thought of the person with ALS contracting COVID-19, consistent with other studies (18, 20).

Increased apathy in pwALS was the most frequently reported type of behavioral change over the pandemic period; the pandemic disrupted routine activity and pwALS demonstrating apathy appeared uninterested in finding something new to do. This finding is unsurprising given that apathy is the most common behavioral change in pwALS (21). In addition to apathy and reduced interest in activities, loss of insight and egocentric behavior in pwALS were reported here, as elsewhere (22). The three participants who reported behavioral change in the person with ALS did not believe that the pandemic had affected behavioral deterioration despite noticing the behavioral change during the pandemic. This may be because lockdown restrictions and shielding behaviors changed their lifestyles, such as interrupting regular hobbies and increasing time at home with their spouse. Therefore, spouses may have had more opportunities to notice behavioral symptoms during the pandemic, although they may have also noticed these had the pandemic not occurred. Given that during this period, access to clinical support was restricted as appointments were canceled and digital alternatives to face-to-face clinical assessments fell short for some participants, describing them as rushed and doubting the ability of clinicians to make thorough assessments, it will be important for clinicians to ask about cognitive and behavioral change in pwALS during future remote as well as face-to-face appointments and offer appropriate support.

Other key findings included increases in caregiver duties, increases in burden, loss of support, and a reduction in opportunities to take a break from the caregiver role (for those who reported having caregiving duties; Table 1) during the pandemic, as also found reported elsewhere (1, 11, 18–20, 23).

### Strengths and limitations

This is one of the first qualitative interview studies of the effects of the coronavirus pandemic on spouses of pwALS in the United Kingdom and is the first to enquire about cognitive and behavioral change in pwALS within the context of spouse experience during the pandemic.

This study has some limitations. The generalizability of findings is limited for a few reasons. Firstly, the final sample of nine participants is not large but is consistent with appropriate sample sizes for thematic analysis (24) and saturation of themes was reached. In addition, ALS may have been less severe in the patients whose spouses were included since the relevant pwALS had participated in another study by these authors, having to be able to communicate clearly orally or to write/type clearly. Consequently, this sample of spouses may have experienced different care duties to those of less independent pwALS with advanced mobility and communication difficulties, which appears to be the case given that 6/9 spouses provided caregiving for less than one hour per week. It would have been informative to have been able to describe the pwALS in terms of disease stage (25) and explore how this might have impacted responses to the pandemic and lockdowns, but this information was not available. However, disease duration of the pwALS varied (Table 1) and caring duties were more likely to be reported by the spouses where disease duration was longer.

While none of the pwALS related to this study had received a clinical diagnosis of cognitive or behavioral impairment or ALS-FTD, some participants were nonetheless spouses of pwALS who met cutoff scores for impairment on at least one of three cognitive screening tools (3/9 participants; Table 2) or at least one of five behavioral screening tools (5/9 participants; Table 2).

All participants had experienced the first lockdown in England and had experienced restrictions being lifted. However, three experienced additional lockdowns prior to being interviewed, two of whom were interviewed during a lockdown (P7, P8, Figure 1). Therefore, subsequent experiences and effects of delayed recall may have influenced their accounts.

Our study has identified specific behavioral changes in pwALS, such as increased apathy, reduced interest in activities, loss of insight, and egocentric behavior, for which caregivers may require support. Clinicians should acknowledge spouses’ concerns about remote consultations in adapting to the changing healthcare delivery landscape, actively enquire about cognitive and behavioral changes, and ensure that caregivers are aware of available support services, particularly in terms of respite care to alleviate feelings of burden. In conclusion, our findings suggest areas for improvement in service delivery in times of future national health crises and aspects of carers’ experiences that should deserve attention and support.

## Supplementary Material

Supplemental Material

## Data Availability

Qualitative interview data cannot be made publicly available due to the possible risk of compromising participants’ confidentiality.
