# Supplementary material for: How the coronavirus pandemic affected the lives of people with ALS and their spouses in the UK from spouses’ perspectives: a qualitative study
Source: Amyotroph Lateral Scler Frontotemporal Degener. 2024 May 8;25(5-6):625–33. doi: 10.1080/21678421.2024.2346501 (PMC11098060; doi:10.1080/21678421.2024.2346501)
Supplement: Supplemental Material [file IAFD_A_2346501_SM4714.zip › Supplementary Information 1 Interviewer Characteristics March 2024.docx]

**SUPPLEMENTARY INFORMATION 1**

**Study title: How the coronavirus pandemic affected the lives of people with ALS and their spouses in the UK from spouses’ perspectives: A qualitative study**

**Lyndsay Didcote^1^, Ammar Al-Chalabi^2,3^ & Laura H. Goldstein^1*^**

**1=King’s College London, Department of Psychology, Institute of Psychiatry, Psychology and Neuroscience, London, UK**

**2=King’s College London, Maurice Wohl Clinical Neuroscience Institute, Department of Basic and Clinical Neuroscience, London, UK**

**3=Department of Neurology, King’s College Hospital NHS Foundation Trust, London, UK**

***Corresponding author Professor Laura H Goldstein Department of Psychology, Institute of Psychiatry, Psychology and Neuroscience, De Crespigny Park, London SE5 8AF laura.goldstein@kcl.ac.uk**

**Interviewer characteristics**

At the time of this study, LD, a female PhD student, was conducting other research into cognitive and behavioural change in pwALS and the impact of this on caregiver distress (anxiety, depression, and burden) and so had an interest in these topics which may have influenced interpretation of the data. She had undergone recent training in qualitative methods. Participants knew that they would be contributing to a study for LD’s PhD. As a result of this ongoing research, LD was familiar with the physical limitations of ALS and the vulnerability of pwALS to COVID-19 and so expected these factors to affect spouses when conducting this study. Furthermore, given the nature of the pandemic and resulting lockdowns, it was reasonable to expect that that spouses of pwALS would experience an increase in isolation and caregiving duties for pwALS who were in need of care. Therefore, an increase in caregiver distress was expected by LD which may also have influenced interpretation of the data.
