# Supplementary material for: How the coronavirus pandemic affected the lives of people with ALS and their spouses in the UK from spouses’ perspectives: a qualitative study
Source: Amyotroph Lateral Scler Frontotemporal Degener. 2024 May 8;25(5-6):625–33. doi: 10.1080/21678421.2024.2346501 (PMC11098060; doi:10.1080/21678421.2024.2346501)
Supplement: Supplemental Material [file IAFD_A_2346501_SM4714.zip › Supplementary Information 2 .docx]

**SUPPLEMENTARY INFORMATION 2**

**Study title: How the coronavirus pandemic affected the lives of people with ALS and their spouses in the UK from spouses’ perspectives: A qualitative study**

**Lyndsay Didcote^1^, Ammar Al-Chalabi^2,3^ & Laura H. Goldstein^1*^**

**1=King’s College London, Department of Psychology, Institute of Psychiatry, Psychology and Neuroscience, London, UK**

**2=King’s College London, Maurice Wohl Clinical Neuroscience Institute, Department of Basic and Clinical Neuroscience, London, UK**

**3=Department of Neurology, King’s College Hospital NHS Foundation Trust, London, UK**

***Corresponding author Professor Laura H Goldstein Department of Psychology, Institute of Psychiatry, Psychology and Neuroscience, De Crespigny Park, London SE5 8AF laura.goldstein@kcl.ac.uk**

**Methods**

Purposive sampling method

Purposive sampling techniques were initially employed, with email invitations first being sent to 10 specific participants. Initially, cognitive and behavioural screening tool scores of pwALS, determined in a related study conducted by the authors, were reviewed; the spouses of pwALS were selected and invited to participate based on these scores, in order to obtain a sample of spouses of pwALS with a range of ALS-related cognitive and behavioural involvement as measured on these screening tools. As spouses declined to participate or did not reply to the invitation, further spouses of pwALS were invited to participate and it was less possible to ensure such a wide range of cognitive and behavioural change in the pwALS.

Field notes and pilot interview

Field notes were recorded after interviews as a method of tracking contextual information. The first interview was conducted as a pilot interview (and was included in thematic analysis) to test the software used to conduct the interviews and obtain participant feedback on the content, structure, and methodology of the interview.

Guiding interviews

Interviews began with questions about background information (e.g., how many people shared the home and what was the quality of the person with ALS’ communication) which helped to develop rapport before more in-depth, personal questions were asked.

Prompts were used when participants were not sure how to answer interview questions. When participants gave accounts of their lives during the pandemic, they were asked to clarify whether they felt that the pandemic had caused what they were describing and whether, if applicable, the subject of their description was a change from before the pandemic. Where interesting content arose during the interview (e.g., specific mentions of pandemic and/or ALS-related topics that were not included in the topic guide, such as cancellation of holidays that were of particular importance to participants because of a sense of limited time due to having ALS), the researcher asked participants to expand on these topics. This information was included in the data analysis.

Eight of nine participants were alone but one participant was unable to be separated from their spouse at the time of the interview.

Thematic Analysis

Thematic analysis was selected primarily because the aim was to summarise the themes contained in the qualitative data. The purpose was not to form a narrative, to analyse language, to explore the data or develop a theory based on this (as in narrative analysis, discourse analysis or grounded theory (1–3)). Instead, the purpose was to identify and provide a detailed account of elements caused by the coronavirus pandemic that contributed to developments specified in the topic guide, in a clear and organised manner (4,5). Furthermore, thematic analysis is particularly good at highlighting similarities and differences in experiences between data given by participants; this is useful in determining which pandemic-related factors contributed to difficulties experienced by pwALS and spouses (5).

Interviews were transcribed verbatim by LD and anonymised during transcription. Transcripts were checked against recordings for accuracy and were read repeatedly by LD to aid familiarisation with the material. Initial codes were identified in all nine transcribed interviews by LD; initial codes were separately identified by LG in three randomly-selected transcribed interviews and the two sets of codes were then compared. Most quotes were coded similarly by both analysts and any differences were noted, discussed and included in the thematic analysis. The remainder of the analysis was undertaken by LD. Coding was undertaken using NVivo (release version 1.6.1 (6)). Coded data included material that was not necessarily related to questions in the topic guide and both negative and positive quotes (e.g., quotes suggesting the pandemic had not increased depression in spouses as well as quotes that suggested that it had).

Patterns in the data were identified by grouping codes into subthemes. New codes and subthemes were added as interviews were analysed. Subthemes were reviewed following analysis of all interview transcripts and subthemes were then grouped into superordinate themes. Saturation of themes was reached; no new major codes were identified in the final two interviews to be analysed.

Superordinate themes, subthemes, codes and example quotations were reviewed with LG and possible changes were discussed and adjustments made.

Limitations of our methodology

We did not ask participants to check the accuracy of transcripts and did not send data summaries to participants for validation, but our data are generally consistent with other research findings.

References

1. Barkhuizen G, Benson P, Chik A. Narrative Inquiry in Language Teaching and Learning Research. 1st ed. New York: Routledge; 2013.

2. Johnstone B. Discourse Analysis. 3rd ed. Hoboken: Wiley Blackwell; 2018.

3. Oktay, J S. Grounded Theory. 1st ed. Oxford: Oxford University Press; 2012.

4. Braun V, Clarke V. Using thematic analysis in psychology. Qual Res Psychol. 2006;3(2):77–101.

5. Nowell LS, Norris JM, White DE, Moules NJ. Thematic Analysis: Striving to Meet the Trustworthiness Criteria. Int J Qual Methods. 2017 Sep 28;16(1). Available from: https://us.sagepub.com/en-us/nam/open-access-at-sage

6. QSR International Pty Ltd. Nvivo qualitative data analysis software. Doncaster, Australia; 2016.
