# Supplementary material for: How the coronavirus pandemic affected the lives of people with ALS and their spouses in the UK from spouses’ perspectives: a qualitative study
Source: Amyotroph Lateral Scler Frontotemporal Degener. 2024 May 8;25(5-6):625–33. doi: 10.1080/21678421.2024.2346501 (PMC11098060; doi:10.1080/21678421.2024.2346501)
Supplement: Supplemental Material [file IAFD_A_2346501_SM4714.zip › Supplementary Information 3 Topic guide March 2024.docx]

**SUPPLEMENTARY INFORMATION 3**

**Study title: How the coronavirus pandemic affected the lives of people with ALS and their spouses in the UK from spouses’ perspectives: A qualitative study**

**Lyndsay Didcote^1^, Ammar Al-Chalabi^2,3^ & Laura H. Goldstein^1*^**

**1=King’s College London, Department of Psychology, Institute of Psychiatry, Psychology and Neuroscience, London, UK**

**2=King’s College London, Maurice Wohl Clinical Neuroscience Institute, Department of Basic and Clinical Neuroscience, London, UK**

**3=Department of Neurology, King’s College Hospital NHS Foundation Trust, London, UK**

***Corresponding author Professor Laura H Goldstein Department of Psychology, Institute of Psychiatry, Psychology and Neuroscience, De Crespigny Park, London SE5 8AF laura.goldstein@kcl.ac.uk**

**Topic and Interview Guide**

Summarising statement about the project and the purpose of the interviews by the interviewer.

Participant is reminded that they do not need to answer the questions if they do not want to, they should inform the interviewer if they are not comfortable discussing a topic.

Participant is reminded that the interview will be recorded. Begin recording once the participant has given their consent for the interview to be recorded.

Once recording, ask the participant to confirm that they have given their permission for the interview to be recorded.

Some questions may be phrased in the past tense depending on whether shielding and government restrictions have ended.

Section 1: Background Information (to develop rapport and obtain context for the content of the remainder of the interview)

- What is your relationship to (the person with ALS)?
- How long have you known (the person with ALS)?
- Do you live in the same household as (the person with ALS)?
- How long ago did they begin noticing symptoms?
- How long ago did they receive their diagnosis?
- Where did their symptoms begin? (Prompt: limb or speech?)
- How is (the person with ALS’) mobility currently?
- What is the quality of (person with ALS’) communication? (Prompt: Is their speech affected? Do they use iPad communication or any other technological assistance?)
- What caring duties do you perform?
- How many hours per week are you undertaking caring duties?
- How many people are living in the household?
- If at all, how do other people in the household contribute to caring for (person with ALS)?

Section 2: Lockdown Experience

- Question 1: Tell me about how, if at all, the people in your household are now protecting themselves and each other as a consequence of the coronavirus pandemic.
  - Is (the person with ALS) shielding or self-isolating currently?
  - How does this differ if at all from what they were doing at the height of the pandemic?
  - Are you and others who live in the household currently isolating with them?
  - How does this differ if at all from what you were doing at the height of the pandemic?
  - At what point in time did (the person with ALS) start shielding/ self-isolating?
  - To what extent are you all observing isolation practices? How if at all has this changed over time?
- Question 2: What has the pandemic been like for (the person with ALS) considering their relatively increased risk status?
  - What are the limitations in everyday life when (the person with ALS) is shielding or self-isolating?
  - How has lockdown changed your daily routines, if it all?
  - How have medical and community support and appointments changed for (person with ALS), if at all?
  - Have your caring duties increased as a result? And those of others?
- Question 3: What elements of daily life have stayed the same or improved over the lockdown period?
  - How has the lockdown changed how you and (the person with ALS) socialise in person and digitally with other people?
  - How has exercise changed during lockdown for you and (the person with ALS)?

Section 3: Impact on Carers

- Question 4: What are your experiences of lockdown as a carer?
  - How has the number of caring duties you are responsible for changed?
  - How has the time you spend doing caring duties changed?
  - How have your caring duties changed, specifically regarding providing company or emotional support or support in terms of mental health for (the person with ALS)?
- Question 5: How, if at all, has the pandemic made you feel more anxious and to what extent has this affected you?
  - How, if at all, has the pandemic altered your employment status and to what extent has this affected you?
  - What are your concerns, if anything, regarding the risks of infection for (the person with ALS) and to what extent has this affected you?
  - What are your concerns, if anything, about becoming infected with the coronavirus yourself and to what extent has this affected you?
  - How well do other people you live with or people outside your household respect your isolation practices? How does this make you feel?
  - How do you feel about leaving the house?
  - How frequently are you and (the person with ALS) checking the news and how does it make you feel? Has this changed as the pandemic has progressed?
  - How do you feel about (the person with ALS) leaving the house?
  - How do you feel about obtaining necessities such as food supplies?
  - How has this anxiety affected you?
  - How difficult have these problems made it for you to do your work, take care of things at home or get along with other people?
  - To what extent do you feel that this anxiety is due to the coronavirus pandemic rather than just being a carer?
- Question 6: How, if at all, has the pandemic made you feel depressed and to what extent has this affected you?
  - How, if at all, has your energy levels changed during the coronavirus pandemic?
  - How has your interest in doing things changed, if at all?
  - How has your appetite changed, if at all?
  - How has your sleeping changed, if at all?
  - If at all, how has how you feel about yourself changed?
  - If at all, how has your ability to concentrate changed?
  - How difficult have these problems made it for you to do your work, take care of things at home or get along with other people?
  - To what extent are these problems due to the coronavirus pandemic rather than just being a carer?
- Question 7: If at all, to what extent has the pandemic changed the feeling of burden you experience as a carer?
  - During the pandemic, to what extent are you able to step outside of your caring role and have some time for yourself?
  - During the pandemic, to what extent do you feel stressed over balancing caring and other responsibilities?
  - To what extent has being a carer during the pandemic changed your relationships with other people?
  - To what extent are you content that you are doing enough for (the person with ALS) and how has this changed during the pandemic, if at all?

Section 4: Impact on pwALS

- Question 8: To what extent, if at all, have any of (the person with ALS’) MND symptoms progressed more rapidly during lockdown compared to symptom progression rate before lockdown? (e.g. communication, mobility)
- Question 9: Does (person with ALS) have a diagnosis of behavioural or cognitive impairment or frontotemporal dementia?
- Question 10: If at all, how has (person with ALS’) thinking changed since lockdown?
  - To what extent does (person with ALS) understand what is happening regarding the pandemic?
  - To what extent does (person with ALS) understand the need to self-isolate or shield?
  - To what extent does (person with ALS) retain information about the pandemic?
  - How well has (person with ALS) been organising themselves on a daily basis?
- Question 11: How has (person with ALS) been behaving since lockdown began? Have there been any changes in their behaviour?
- Question 12: If there have been any changes in thinking or behaviour, to what extent has lockdown influenced these changes?
  - If there have been any changes in thinking or behaviour, how do you think that lockdown has affected the rate at which (person with ALS) has deteriorated in terms of changes in their thinking and behaviour?
- Question 13: If there have been any changes in thinking or behaviour, has this made dealing with lockdown more difficult?
  - In what way?
  - If there have been any changes in thinking or behaviour in the (person with ALS), to what extent has this contributed to your feelings of anxiety or depression?

End:

- Is there anything else you would like to add that you think might be relevant to the things that I have asked you about?
- If you have any concerns about anything you have told me about, you should contact your GP or the MND clinic/neurologist.
