# Supplementary material for: How the coronavirus pandemic affected the lives of people with ALS and their spouses in the UK from spouses’ perspectives: a qualitative study
Source: Amyotroph Lateral Scler Frontotemporal Degener. 2024 May 8;25(5-6):625–33. doi: 10.1080/21678421.2024.2346501 (PMC11098060; doi:10.1080/21678421.2024.2346501)
Supplement: Supplemental Material [file IAFD_A_2346501_SM4714.zip › Supplementary Information 4 Themes and Codes.docx]

# SUPPLEMENTARY INFORMATION 4

# **Study title: How the coronavirus pandemic affected the lives of people with ALS and their spouses in the UK from spouses’ perspectives: A qualitative study**

Lyndsay Didcote1, Ammar Al-Chalabi2,3 & Laura H. Goldstein1*

1=King’s College London, Department of Psychology, Institute of Psychiatry, Psychology and Neuroscience, London, UK

2=King’s College London, Maurice Wohl Clinical Neuroscience Institute, Department of Basic and Clinical Neuroscience, London, UK

3=Department of Neurology, King’s College Hospital NHS Foundation Trust, London, UK

*Corresponding author Professor Laura H Goldstein Department of Psychology, Institute of Psychiatry, Psychology and Neuroscience, De Crespigny Park, London SE5 8AF laura.goldstein@kcl.ac.uk

# Subthemes that may apply to the general population, other vulnerable groups or to pwALS if the pandemic had not occurred

*Pandemic behaviours: cautious behaviour*. These experiences may be similar to those of the general population or other vulnerable groups. Participants frequently described their use of face masks and hand sanitiser in preventing the spread of COVID-19, their avoidance of going outside, their reduction of face-to-face contact and their initial behaviours at the beginning of the pandemic. Three participants also gave a self-assessment of their level of caution (see Table 1).

When asked about how they protected themselves and the person with ALS from contracting COVID-19, six participants spoke about wearing face masks in shops, sometimes prior to government advice and sometimes only after it was legally required. Using hand sanitiser, washing their hands, not touching their face, and social distancing were also reported. One participant spoke about how they reminded the person with ALS to wear a face mask and sanitise their hands when they went out.

The avoidance of going outside by the person with ALS and their spouse included a reduction in going for walks, restaurant visits, and going into shops (when restaurants and shops were open during the pandemic). Spouses also reported that the person with ALS did not enter any buildings outside their home and one stopped going to the gym before gyms were legally required to close. The avoidance of shops was also raised when participants described reducing face-to-face contact. Participants additionally mentioned limiting contact with others to at the supermarket only, asking for deliveries to be left on the doorstep, and not allowing others indoors.

When asked about the beginning of the pandemic, participants primarily spoke about shielding behaviours including beginning to work from home and leaving work to reduce exposure to COVID-19. Some families merged households (i.e., adult children of the pwALS and/or their spouse moved in with their parent(s)) at the beginning of the pandemic so that they could isolate together (see quote 1.1.4.2, Table 1). One participant described cleaning the packaging of food products that entered the house (see quote 1.1.4.3, Table 1).

Participants described themselves as careful, more so than others, and as taking all precautions possible. However, one participant said that their methods of protecting themselves were not perfect. Another scored their level of caution as “eight out of ten” (see quote 1.1.5.3, Table 1).

*Pandemic behaviours: relaxation of caution.* These experiences may be similar to those of the general population or other vulnerable groups. Participants described relaxation of their cautious behaviour after lockdowns by beginning to go outside more and developing more of a balanced approach between living freely and preventing infection. Four participants reported that they and the person with ALS that they cared for did not engage in any shielding behaviour at any time. A number of spouses relaxed their level of caution by being outdoors more or going further than before. Most were still not comfortable going indoors after lockdown restrictions were relaxed but some spoke about going to the hairdressers, going into restaurants and attending socially distanced live music events.

Reasons for not shielding or having a more balanced approach to protecting themselves were primarily about wanting to have normal life experiences. They included feeling the need to socialise, needing to go out and it being good for them, and some more extreme cautious behaviour (i.e., cleaning food packaging from supermarkets) became tiring. One participant reported not feeling anxious at any point of the pandemic and felt there was an inevitability to contracting COVID-19.

*Pandemic behaviours: others’ attitude to shielding pwALS.* These experiences may be similar to those of other vulnerable groups. Most interactions with others were positive, with participants feeling that their shielding practices were respected. Friends often recognised that because their friend had ALS or lived with someone with ALS, they had to be particularly careful. For example, one friend socialised with a person with ALS through a closed door. Two spouses felt that friends were not particularly cautious around the person with ALS because their ALS symptoms were not very noticeable. One participant had to set boundaries with friends and felt guilty about this but also felt it was necessary (quote 1.3.1.4, Table 1).

Mostly, spouses reported that trade workers were careful about not spreading COVID-19 by wearing facemasks and gloves, and by taking routes through the house that avoided main living areas. However, one spouse reported having to request these measures and this caused them to feel angry (see quote 1.3.2.2, Table 1).

The organisations that participants spoke about when discussing others’ attitudes to their needing to shield were the NHS and government-supermarket food delivery arrangements. Three participants reported that the person with ALS received an NHS letter informing them of their vulnerable status and their need to shield; however, one participant reported contracting COVID-19 before receiving this letter and two others did not receive a letter at all. One complaint was made about government-assisted supermarket deliveries for the vulnerable and a further complaint was made about not being prioritised for COVID-19 vaccination

| **Table 1: Summary of identified subthemes and codes related to pandemic behaviours with illustrative quotations** | | |
| --- | --- | --- |
| Subthemes | Codes | Example quotes |
| Cautious behaviour^†‡^ | Masks and sanitiser | 1.1.1.1 “I always check I have my mask, my hand sanitiser.” P1 |
|  |  | 1.1.1.2 “I have worn a mask and hand sanitiser” P5 |
|  |  | 1.1.1.3 “washing hands, wearing masks, not touching stuff, no touching your face.” P9 |
|  | Avoiding going outside | 1.1.2.1 “we just don’t go out as much as we used to… at the weekend we would go out and eat a lot, go out and walk a lot. So all of that is gone.” P7 |
|  |  | 1.1.2.2 “we're not making unnecessary trips to the particular things like the shops and whether it might be a lot of people and surfaces” P2 |
|  | Reducing F2F contact | 1.1.3.1 “Any deliveries to the house, I still ask them to leave them on the doorstep.” P1 |
|  |  | 1.1.3.2 “we don’t mix.” P3 |
|  | Beginning of pandemic behaviour | 1.1.4.1 “he was more at risk. So I stopped work a week before the end of term” P6 |
|  |  | 1.1.4.2 “our son came home just before it all hit” P8 |
|  |  | 1.1.4.3 “everything that was coming in, I was literally spraying everything” P2 |
|  | Self-assessment of caution | 1.1.5.1 “we are more careful than most people” P7 |
|  |  | 1.1.5.2 “we’re just doing everything we can and you know taking all the precautions” P2 |
|  |  | 1.1.5.3 “overall we have probably been probably eight out of ten good.” P8 |
| Relaxation of caution^†‡^ | Going outdoors | 1.2.1.1 “We did start being a bit bolder around the second lockdown and we did go out to restaurants a couple of times, we went to listen to some live music once” P7 |
|  |  | 1.2.1.2 “Whereas now we would go to PLACE which is a busy beach car park.” P1 |
|  |  | 1.2.1.3 “I'm going to the hairdressers” P4 |
|  | Not shielding/ balanced approach | 1.2.2.1 “we have got to the point now where they don’t bother (laughing) they just come in and sit down” P3 |
|  |  | 1.2.2.2 “they always say well we have got a bit of a sniffle or a cold … and we say yeah come on down” P3 |
|  |  | 1.2.2.3 “there are couple of times where probably encouraged by drink we've been a little bit more tactile or you know in closer proximity approximately than we were, otherwise we probably should have been”. P8 |
|  |  | 1.2.2.4 “I feel we've got to we've got to live.” P6 |
| Others’ attitude to shielding pwALS^‡^ | By friends | 1.3.1.1 “They are friends that know our situation so they were still social distanced, they were very conscious of not touching things” P6 |
|  |  | 1.3.1.2 “our friends won’t mix they are frightened of giving it to PATIENT” P3 |
|  |  | 1.3.1.3 “Pretty well, well we got friends who are a little bit more relaxed about it and trying to batter your front door down” P8 |
|  |  | 1.3.1.4 “Slightly guilty I suppose or or such but not guilty that… we're rejecting and that we we don't like to hurt people's feelings… I'm not sure that they think of PATIENT as being vulnerable” P8 |
|  | By strangers | 1.3.2.1 “And we knew that the people, the gardener and the trainer, we knew that they were also respecting us and also themselves, they were taking care.” P5 |
|  |  | 1.3.2.2 “where they haven't maybe worn a mask I remember asking somebody, you know, ‘what are you doing?’” P4 |
|  | By authorities | 1.3.3.1 “the medical authorities don't seem to have regarded her to be a risk.” P8 |
|  |  | 1.3.3.2 “I think cos PATIENT had a letter to say he must shield… an NHS letter. So … possibly it was then.” P5 |
|  |  | 1.3.3.3 “I was desperate to try and… get all the shopping delivered but couldn't and even though I've gone on the government website… it just didn't seem to count or make a difference” P2 |

Ellipses indicate where words have been omitted from the quotation for the purpose of representing the meaning of the quote within a limited space. F2F = Face-to-face. P = participant number. pwALS = people with Amyotrophic Lateral Sclerosis. ^†^ = These experiences may be similar to those of the general population. ^‡^ = These experiences may be similar to those of other vulnerable groups.

*Changes to daily life caused by the pandemic and progression of ALS: changes due to ALS progression.* Changes to the daily life of the person with ALS, as a result of the progression of their ALS over the pandemic period, was reported by spouses. Spouses also described how this had changed their daily life (see Table 2). The changes in daily life for pwALS that spouses spoke about were primarily concerned with reduction in experiences and hobbies due to ALS (e.g., not being able to eat at restaurants due to embarrassment caused by dysphagia; it being unsafe to continue with a water sport). For spouses, changes were relevant to physical symptoms of ALS (e.g., having to move to an adapted house; preparing activities to do within the home in the future because they anticipated not being able to leave the house; see quote 2.1.2.3, Table 2 for example). Participants are likely to have reported these experiences had the pandemic not occurred.

*Changes to daily life caused by the pandemic and progression of ALS: changes due to the coronavirus pandemic*. These experiences may be similar to those of other vulnerable groups. The coronavirus pandemic was said to have altered the number or duration of caring duties, and medical appointments for the person with ALS. The pandemic increased caregiving duties in several ways. The pandemic led to adult children returning to live at their parents’ home, which increased chores such as cleaning, cooking, and food shopping; for example, see quote 2.2.1.1, Table 2. The pandemic also increased the need for spouses to entertain the person with ALS, and also prevented them from eating out (increasing cooking chores). One spouse was particularly distressed over the disruption of occupational therapy support as it left them feeling unqualified to be a caregiver and needing to do research. As a result, they felt that their caregiving duties took longer than if they had received professional advice (see quote 2.2.1.3, Table 2). However, most participants said the pandemic had not increased caring duties, but some expressed a lack of being able to step outside of their caring role and do something for themselves or receive care from others.

Eight out of the nine participants reported an increase in digital and a reduction in face-to-face communication with friends and family (often reducing meetings as much as by several times per week). This was felt to have strained relationships and left participants feeling lonely. However, three participants had a substantial increase in face-to-face contact with others over the pandemic period because adult children moved into their home. Six participants expressed a preference for in-person social interactions. Two participants described digital meetings as difficult to orchestrate and one expressed that phone etiquette prevented them from being open about their feelings and seeking social support.

| **Table 2: Summary of identified subthemes and codes related to changes to daily life caused by the COVID-19 pandemic and progression of ALS with illustrative quotations** | | |
| --- | --- | --- |
| Subthemes | Codes | Example quotes |
| Changes due to ALS progression* | For the person with ALS | 2.1.1.1 “he would definitely not go into a restaurant now because of his um dysphagia anyway, it would be too stressful and embarrassing.” P1 |
|  |  | 2.1.1.2 “we would have sailed a race 3 times a week. And because of, at the start of PATIENT’s illness his arm weakness went so we realised it wasn’t safe to sail. P1 |
|  |  | 2.1.1.3 “he hasn't cycled this year at all and he's realised that he can't cycle anymore so...” P4 |
|  | For the spouse | 2.1.2.1 “it was our flat and obviously I had maintenance work to do… it was getting to the point where PATIENT said ‘look this is no holiday at all… we will have to sell the flat’.” P3 |
|  |  | 2.1.2.2 “I think probably I spend more time on my own perhaps when he sleeps more so I am waiting for him to wake up” P1 |
|  |  | 2.1.2.3 “I have bought a rowing machine so not just for lockdown thinking forward when the disease progresses and I can’t go out as much.” P1 |
| Changes due to COVID-19 pandemic^‡^ | Change in caregiving duties | 2.2.1.1 “I think um obviously we've got a lot of people working from home… there's a lot to do around the house and the shopping.” P6 |
|  |  | 2.2.1.2 “there is more that I have to do than I did at the start of lockdown but it is difficult to separate what is the effect of lockdown and what is the effect of PATIENT… deteriorating.” P7 |
|  |  | 2.2.1.3 “the shower chair and getting out the bath, we work out ourselves. Maybe I have done a little more research than if a professional… was able to tell me exactly, you know, the facts.” P1 |
|  |  | 2.2.1.4 “It is not affected, it it's been pretty good actually.” P4 |
|  | Changes to social interaction | 2.2.2.1 “I am probably am doing a lot more digital contact, yes. My daughter now phones me probably every other day and we do facetime with the grandchildren.” P1 |
|  |  | 2.2.2.2 “we were gonna meet up in PLACE… but because the the COVID levels were rising just at that end… if it was just me, it it would be OK but I risk bringing something back to PATIENT and I I can't, I can't be doing that.” P4 |
|  |  | 2.2.2.3 “I mean we're both very social animals. I would say that in terms of our lives, it's probably one of the most important things … it's it's certainly, it's it's diluted our quality of life.” P8 |
|  |  | 2.2.2.4 “So we did that and now we sort of meet up maybe once a week and we just go for a walk (muffled) and sit which is sort of breaking the rules slightly.” P7 |

Ellipses indicate where words have been omitted from the quotation for the purpose of representing the meaning of the quote within a limited space. P = participant number. ALS = Amyotrophic Lateral Sclerosis. * = Participants are likely to have reported the same experiences had the pandemic not occurred. ^‡^ = These experiences may be similar to those of other vulnerable groups.

*Distress in spouses: anxiety.* These experiences may be similar to those of other vulnerable groups. Spouses reported anxiety over themselves contracting COVID-19 and about being in crowded areas (for example, see quote 3.1.2.2, Table 3). Participants spoke about experiencing anxiety over face-to-face contact with others and being in other people’s houses. Three participants, however, did not express any anxiety or expressed positive views towards the person with ALS leaving the house (for example, see quote 3.1.3.3, Table 3).

| **Table 3: Summary of identified subthemes and codes related to distress in spouses as a result of the coronavirus pandemic with illustrative quotations** | | |
| --- | --- | --- |
| Subthemes | Codes | Example quotes |
| Anxiety^‡^ | About the spouse contracting COVID-19 | 3.1.1.1 “Um and all being ill myself, I just don't think it would be very good idea at all.” P4 |
|  |  | 3.1.1.2 “um going onto the next stage of what would happen to me if I went into hospital… I suppose I would be quite scared.” P5 |
|  | In crowds | 3.1.2.1 “But the number of times we would go out for a walk and somebody would get so close to you that we just… so annoying. And it put us off going for a walk in a way” P7 |
|  |  | 3.1.2.2 “Certainly, as we came out of lockdown, I was very anxious about going into town… there was so many tourists in the town. It was crowded.” P1 |
|  | No anxiety | 3.1.3.1 “But I think because because she had it we we think we're pretty sure she had it so early on, the concern hasn't really been there.” P8 |
|  |  | 3.1.3.2 “We have come to the conclusion that we are all going to get it in the end... There are a lot of people saying “let’s have it, let’s get it over and done with” you know.” P3 |
|  |  | 3.1.3.3 “I guess as it has gone on there has not been that much anxiety really.” P9 |

Ellipses indicate where words have been omitted from the quotation for the purpose of representing the meaning of the quote within a limited space. P = participant number. pwALS = people with Amyotrophic Lateral Sclerosis. ^‡^ = These experiences may be similar to those of other vulnerable groups.
